# Diet plays a supportive role in managing thyroid disorders – but a critical one!

**DOI:** 10.1530/ETJ-26-0185

**Published:** 2026-06-17

**Authors:** Luca Persani, Leonidas H Duntas

**Affiliations:** ^1^ Department of Endocrine and Metabolic Diseases, IRCCS Istituto Auxologico Italiano, Milan, Italy; ^2^ Department of Medical Biotechnology and Translational Medicine, University of Milan, Milan, Italy; ^3^ Evgenidion Hospital Unit of Endocrinology, Diabetes, and Metabolism, Thyroid Section, National and Kapodistrian University of Athens, Athens, Greece

**Keywords:** iodine, selenium, thyroid function, nutrition

## Abstract

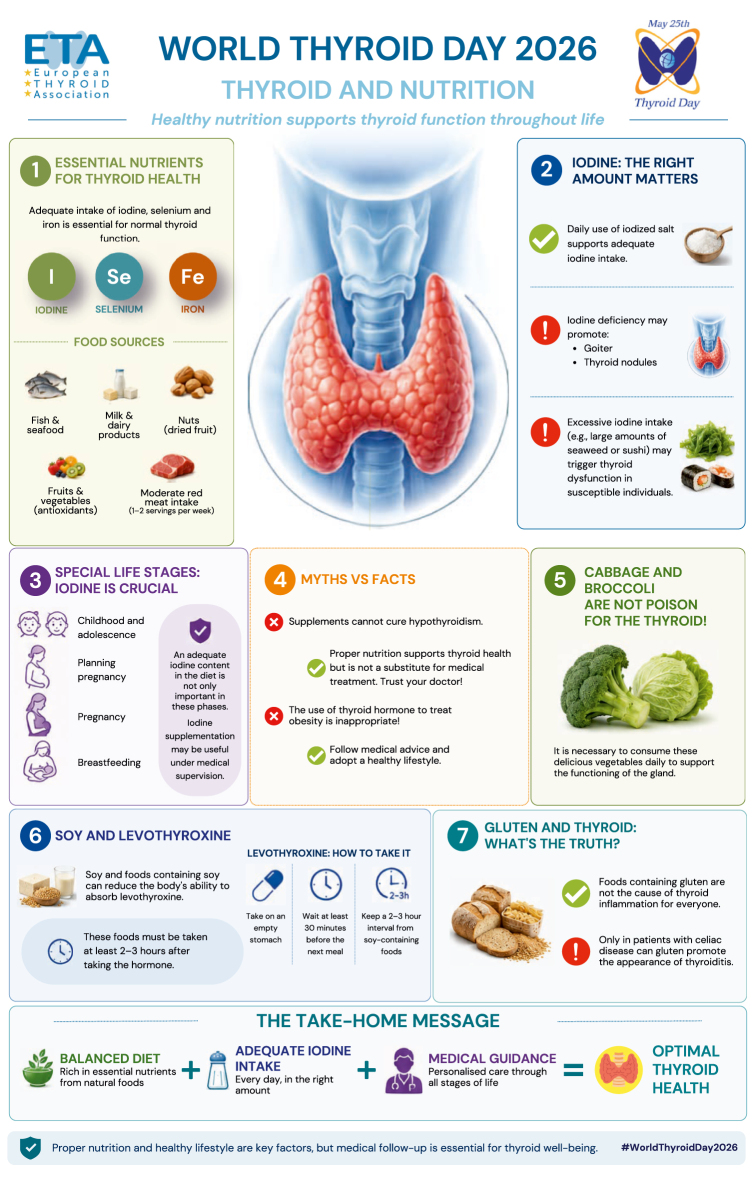

## Nutrition and thyroid function

The thyroid is like a metabolic engine converting nutrients into energy for the entire body. Among the nutrient energy sources are macronutrients, such as carbohydrates, proteins, and fats, which are the primary sources that build and repair muscles and support cellular structure, and micronutrients, which are essential elements for growth, immune function, and disease prevention (1).

Thyroid health also depends on adequate, synergistic intake of iodine, selenium (Se), and iron, known as the critical ‘bodyguards’ of thyroid function. Iodine, typically consumed as iodide in food, enters the circulation and is taken up by the sodium–iodide symporter (NIS) into the thyrocyte, where it is oxidized to iodine by thyroid peroxidase (TPO). Iodine-iodinated tyrosyl residues in thyroglobulin drive the formation and release of thyroxine (T4) and triiodothyronine (T3) into the circulation. Daily sources of iodine include seafood, seaweed, dairy, eggs, and iodized salt, all of which are essential for thyroid health and metabolism.

However, balance is needed. While low dietary iodine intake promotes goiter and possibly thyroid nodules, high amounts taken regularly with meals or with drugs can be toxic to the thyroid and worsen preexisting (unrecognized) forms of hyperthyroidism (Graphical Abstract).

Selenium (Se), a trace mineral the body needs only in tiny amounts to stay healthy, is found in many animal- and plant-based foods (2). The body uses Se to produce 25 proteins (selenoproteins) that play critical roles in protecting cells against damage by activating the glutathione peroxidase (GPX) and thioredoxin reductase (TXNRD) systems, supporting the immune system, and maintaining the healthy function of the thyroid gland through the activity of deiodinases (DIOs) (3). In the thyroid gland, GPX regulates the production of hydrogen peroxide (H2O2), protecting thyrocytes from damage. The unique process of selenoprotein synthesis relies on dedicated molecular machinery in which the incorporation of Se as the amino acid selenocysteine (Sec) requires the translational reprogramming of a UGA stop codon to Sec (4). Se is acquired through the diet, primarily from lean meat, eggs, dairy, fish, grains, vegetables, Brazil nuts, and legumes. After digestion and absorption, Se is metabolized in the liver and transported via the bloodstream by a selenoprotein called selenoprotein P (SELENOP) (5).

Iron is at the core of the TPO enzyme, which oxidizes iodide. Low iron levels can reduce TPO activity, leading to lower production of thyroid hormones and contributing to hypothyroidism or worsening existing thyroid conditions, particularly in pregnancy. Iron deficiency also impairs the conversion of T4 to the active T3 hormone.

Ferritin, the iron-storage protein, is a key marker of total-body iron. Optimal ferritin levels for thyroid function are generally considered to be 70–100 ng/mL. Low ferritin can impair thyroid hormone utilization, reduce red blood cell production, and exacerbate such symptoms as fatigue, hair loss, cold intolerance, and mood disturbances, which are common in hypothyroid patients. Recently, a strong negative correlation between TSH and ferritin levels and a weak negative correlation between anti-TPO (TPOAb) and anti-TG (TgAb) antibodies and ferritin levels were reported in patients with Hashimoto’s thyroiditis (HT) (6). These findings support the hypothesis that hypothyroidism may lead to sideropenia and anemia. Normalizing thyroid hormone levels can improve ferritin without additional iron supplementation, underscoring the vital link between iron and thyroid function.

While hypothyroidism is widely recognized as a contributing factor to these conditions, it is often one of several underlying causes rather than the sole trigger in every patient.

In addition to the three essential microelements for thyroid function, other nutrients, such as zinc and vitamin D3, may be protective when adequate, although conclusive data are lacking on their ability to prevent or treat thyroid disease (6, 7). A recent systematic review and meta-analysis found no difference in B12 levels between patients with subclinical hypothyroidism and those with hyperthyroidism, whereas hypothyroid patients had lower B12 levels than healthy adults (8). These results suggest that the degree of hypothyroidism may affect vitamin B12 metabolism and anemia.

Dietary micronutrients are essential for various biochemical processes, including gene transcription, enzymatic and hormonal reactions, and cellular protection. Proper nutrition, including adequate micronutrient intake, supports thyroid function and health. However, in cases of thyroid dysfunction, nutrition cannot substitute for medical treatment. Moreover, supplements alone cannot cure thyroid disease.

## Siren-call diets and thyroid

The many ‘siren-call’ diets advertised online as ‘thyroid diets’ or ‘friendly diets’ for thyroid disorders inevitably lack a single ‘ideal’ plan that would effectively treat or cure thyroid issues – this ‘hurdle’ applies to all major diseases. While basic healthy eating is essential, personalized nutrition plans are crucial for effective management of these conditions. Nutritional strategies can significantly modulate hormone production, alleviate associated symptoms, such as fatigue and weight fluctuations, and help reduce inflammation in autoimmune thyroid diseases, such as Hashimoto’s or Graves’ disease.

Diet plays a critical supportive role in managing symptoms, optimizing thyroid function, and enhancing the effectiveness of medication.

However, we recommend avoiding an unhealthy, high-energy diet rich in animal fats and proteins, salt, and refined sugars (the so-called western diet) because it increases the risk of autoimmunity by altering immune balance and the composition of the gut microbiota.

In contrast, the Mediterranean diet, thanks to its nutritional components, positively influences immune function, the composition of the gut microbiota, and redox homeostasis, exerting antioxidant, anti-inflammatory, and immunomodulatory effects (9).

Notably, a recent experimental study found that the ketogenic diet (KD) ameliorates iodine-induced AIT in mice by modulating inflammation, restoring immune balance, and reducing thyroid autoimmunity (10). These findings may support the use of KD as an adjuvant therapy for AIT and warrant clinical evaluation.

Conclusively, for many complex and chronic conditions, obviously including thyroid disorders, there is no single ‘best diet’ since individual metabolic responses, genetics, and specific triggers can differ significantly from person to person.

Equally importantly, however, diet plays a supportive role in managing thyroid disorders – but a critical one!

World Thyroid Day (WTD) was founded by the European Thyroid Association (ETA) in 2008. The event is widely celebrated yearly, on May 25, across Europe under the auspices of the ETA, while International Thyroid Awareness Week (ITAW) is coordinated by the Thyroid Federation International.

The focus of WTD 2026 is ‘Thyroid and Nutrition’.

On the occasion of WTD 2026, we have compiled eight answers to frequently asked questions regarding thyroid health, as follows:

- Thyroid health relies on adequate intake of iodine, selenium, and iron! Only iodine taken with food enters the bloodstream and becomes available to the thyroid; moderate daily use of iodized salt helps achieve the iodine level necessary for proper gland function. Low iodine intake can cause goiter and thyroid nodules, whereas regular consumption of high amounts of iodine, such as in meals of seaweed salad or sushi, can be toxic to the thyroid and worsen preexisting hyperthyroidism.
- Among the very best natural dietary sources of iodine and Se are fish and other seafood (three portions per week are recommended). Other optimal foods for the thyroid include dried fruit, milk and dairy products, and antioxidants found in fresh fruit and vegetables. Consumption of 1–2 portions per week of red meat and lentils, which are sources of iron, is advisable.
- Proper nutrition supports thyroid health but is not a substitute for medical treatment. Supplements can also not cure hypothyroidism: trust your doctor!
- Adequate dietary iodine is especially important during growth phases (childhood and adolescence), particularly for children with thyroid disorders. Supplementation with iodine-based preparations is highly recommended for women planning to become pregnant, as well as during pregnancy and breastfeeding. Ask your doctor.
- The use of thyroid hormone to treat obesity among people with normally functioning thyroids is inappropriate!
- It is a common misunderstanding that cabbage and broccoli are toxic to the thyroid: this is not true – unless we speak of excessive amounts in individuals already deficient in iodine! These exceptionally healthy vegetables are recommended to support the gland’s health and function.
- Soy and soy-containing foods can reduce the body’s ability to absorb levothyroxine; these foods must be consumed at least 2–3 h after taking the hormone. Levothyroxine should be taken on an empty stomach. The European Thyroid Association and the American Thyroid Association recommend taking the medication about 60 min before breakfast or at bedtime, at least 3–4 h after dinner. Some drugs and supplements containing calcium and iron can also interfere with thyroxine absorption and should be taken at least 2 h after levothyroxine.
- Foods containing gluten are not a cause of thyroid inflammation for everyone; it is only among patients with celiac disease that gluten can promote the development and progression of thyroiditis.

In conclusion, a varied diet, the correct use of iodized salt, and regular consultation with doctors and specialists help protect thyroid health, thereby contributing to reducing the burden of thyroid diseases, which today affect more than 10% of the general population worldwide.

## Declaration of interest

The authors declare that there is no conflict of interest that could be perceived as prejudicing the impartiality of the work reported.

## Funding

This work did not receive any specific grant from any funding agency in the public, commercial, or not-for-profit sector. 
